# Do not Forget to Measure the Head: Hydrocephalus Can Phenotypically Mimic Developmental Coordination Disorder

**DOI:** 10.1177/08830738241302252

**Published:** 2025-01-09

**Authors:** Martinica Garofalo, Jelte Helfferich, Reina W. Kloet, Deborah A. Sival, Kirsten R. Heineman

**Affiliations:** 1 Department of Pediatric Neurology, 10173 University Medical Center Groningen, University of Groningen, Groningen, the Netherlands; 2 Expertise Center Movement Disorders Groningen, 10173 University Medical Center Groningen, University of Groningen, Groningen, the Netherlands; 3 Department of Radiology, 10173 University Medical Center Groningen, University of Groningen, Groningen, the Netherlands; 4 Department of Developmental Neurology, Beatrix Children's Hospital, 10173 University Medical Center Groningen, University of Groningen, Groningen, the Netherlands

**Keywords:** Arachnoid Cyst, Developmental Coordination Disorder (DCD), Head Circumference, Hydrocephalus

## Abstract

Developmental Coordination Disorder (DCD) is a neurodevelopmental condition presenting with poor motor skill development and impaired coordination at a young age. To diagnose DCD, neurologic conditions explanatory for the phenotype, including structural brain abnormalities like hydrocephalus, must be first ruled out. However, these neurologic conditions may phenotypically mimic DCD, which can hamper their distinction. In this article, we report a patient in whom the initial diagnosis of DCD was withdrawn after the identification of acquired hydrocephalus. An important cue in this case was secondary macrocephaly (from +0.00 to +2.25 standard deviations over approximately 6 years’ time). This case illustrates that, in children whose phenotypes seemingly fulfill the DCD criteria, it is important to rule out an underlying, treatable etiology before making the diagnosis of DCD. Since few structural brain abnormalities mimicking DCD may present with macrocephaly, including hydrocephalus, performing longitudinal head circumference measurements can be useful to timely identify these neurologic conditions.

Developmental Coordination Disorder (DCD) is a neurodevelopmental condition characterized by poor motor skill development and non-progressive motor coordination problems presenting during childhood.^1,2^ To diagnose DCD, the following four descriptive criteria must be met: 1) the acquisition and execution of motor skills are below expectation for the child's age; 2) the motor impairments interfere with the child's daily functioning; 3) the symptoms appear during early development; 4) neurologic disorders explanatory for the symptoms have been ruled out, including conditions affecting movement, intellectual delay and visual impairments.^1,2^ Phenotypically, many neurological conditions affecting movement may fulfill at least the first three criteria, including various musculoskeletal conditions and genetic disorders.^3^ This may challenge the phenotypical distinction of these neurological conditions from DCD (unpublished data, M. Garofalo et al., 2024). Furthermore, several progressive neurological disorders affecting motor coordination can have a stationary phenotype in the early phase of disease presentation, and may thus resemble DCD. These disorders include structural brain abnormalities, like hydrocephalus.^4^ Hydrocephalus is a pathological imbalance in the cerebrospinal fluid homeostasis, associated with ventricular expansion and, often, increased intracranial pressure.^5^ Based on the underlying etiology, hydrocephalus can be defined as congenital or acquired.^5,6^ Congenital hydrocephalus can be caused by genetically determined cerebral and/or spinal malformations, such as Dandy-Walker or Chiari malformations, or spina bifida. Acquired hydrocephalus can result from perinatal intraventricular hemorrhage, brain tumors, or arachnoid cysts.^5,6^ The symptoms of hydrocephalus may vary according to the underlying etiology and age of presentation, and can include signs of an elevated intracranial pressure, such as headaches and vomiting, frontal bossing, hypertonia, and/or (progressive) macrocephaly, defined as 2 standard deviations (SD) above the norm.^7–9^ Poor motor skill development, mainly affecting gross motor skills, can also be observed.^7,8,10^ Because of this, and especially in lack of other neurological signs, the phenotypes of hydrocephalus and DCD could initially be mistaken for one another. Despite this phenotypical similarity, hydrocephalus is not explicitly mentioned in the official guidelines as a neurological condition to rule out before diagnosing DCD.^1,2^ Since surgical treatment is often indicated,^11,12^ it is important to consider a potential diagnosis of hydrocephalus in children with phenotypes resembling DCD.

In this article, we present a patient who initially received the diagnosis of DCD, but after experiencing status epilepticus was eventually diagnosed with acquired hydrocephalus. We highlight the importance of measuring head circumference, along with performing additional neuroimaging examinations, in children with phenotypes resembling DCD to detect a potential underlying diagnosis of hydrocephalus.

## Case Presentation

**Figure 1.**
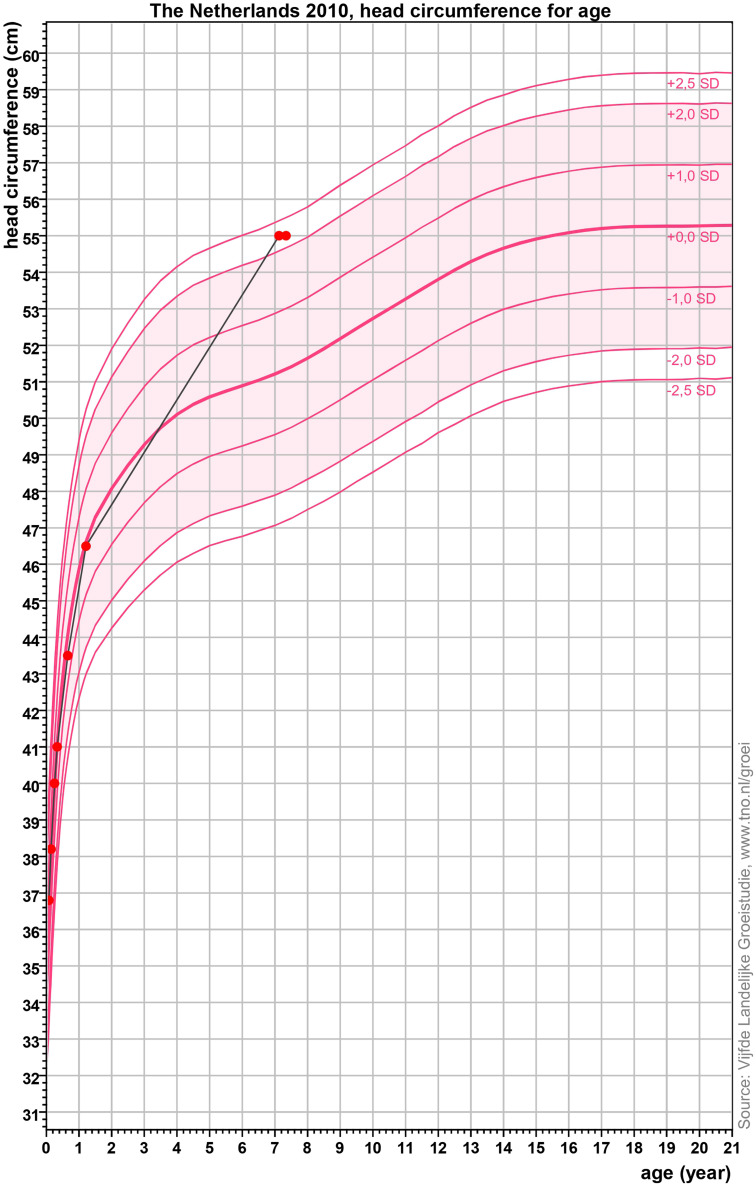
Growth curve of the head circumference for age of the patient reported in this article. The *x* axis shows the age in years, while the *y* axis shows the head circumference in centimeters (cm). Standard deviation (SD) curves are depicted in dark pink and refer to the standard head circumference for age of Dutch children born full term in 2010 (source: Vijfde Landelijke Groeistudie, www.tno.nl/groei). The red dots indicate the head circumference measurements of the patients reported in this article. The last 2 measuring points, performed at the age of 7 years 1 month and 7 years 3 months, amount to 55.018 cm (+2.25 SD). This graph was made using the program Growth Analyser (available at: www.growthanalyser.org).

**Figure 2.**
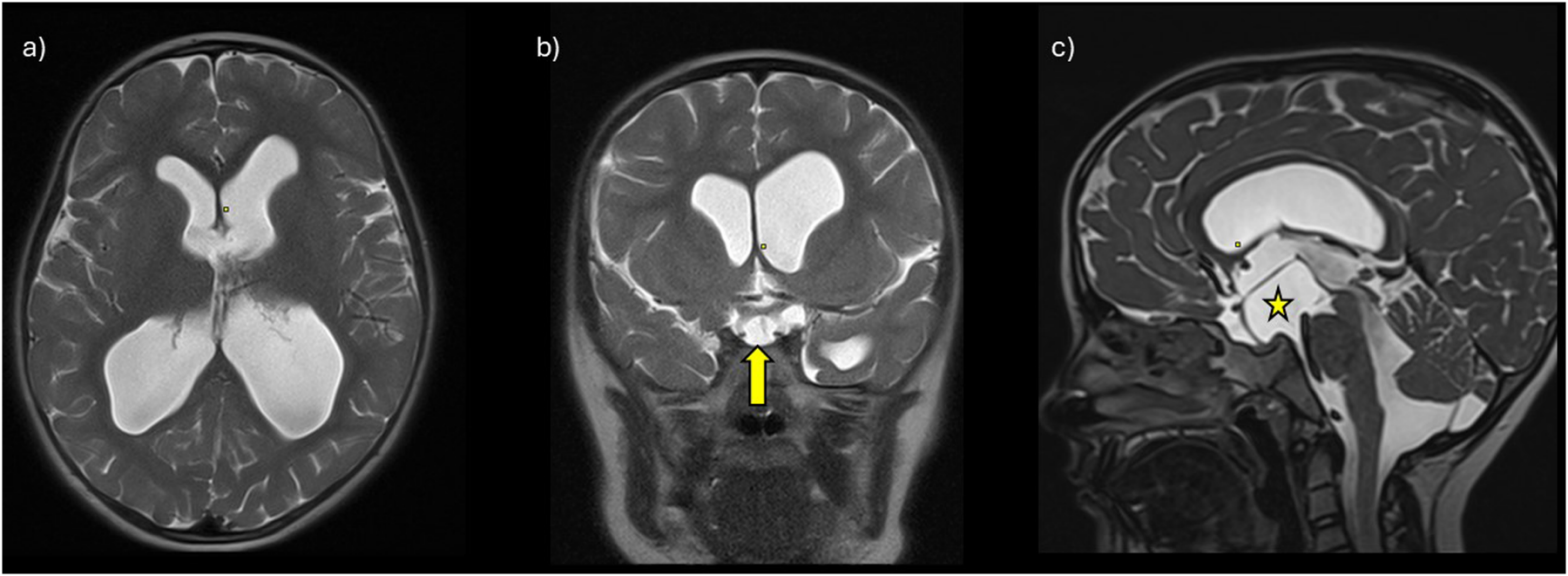
Brain magnetic resonance imaging (MRI) findings of the reported patient. From left to right (a-c): axial, coronal, and sagittal T2-weighted data. Different magnetic resonance imaging (MRI) sequences were used (turbo spin-echo [TSE] in a and b, SPACE [Sampling Perfection with Application optimized Contrast using different flip angle Evolution] in c). Ventriculomegaly is visible in all planes (a-c). The yellow arrow and the star indicate the arachnoid cyst in the interpeduncular cistern (b-c), with mass effect on the optic chiasm and optic nerves (b), and on the pituitary stalk, mamillary bodies, and the midbrain (c). Flow artifacts of the cerebrospinal fluid can be seen in the foramen of Monro on both sides (a) and in the cerebral aqueduct (c).

A 7-year-old girl was referred to the Pediatric Neurology Outpatient Clinic of the University Medical Center Groningen (UMCG) after having experienced status epilepticus. She was born full term after an uncomplicated pregnancy. Family history was negative for neurological disorders. The parents reported that, in infancy, her gross motor development had been on time, but qualitatively suboptimal. For example, she walked independently by the age of 18 months, but her walking pattern was stiff, and she often fell. Her fine motor and language development were unremarkable, and her cognition was unimpaired. Until the age of 15 months, head circumference measurements performed at the child healthcare clinic were normal (+0.00 SD). At the age of 3 years, child physiotherapy was started because of her gross motor problems. For the same reason, at the age of 6 years, she was evaluated by the physiatrist. Because of the lack of objective neurological impairments and on fulfillment of the DCD criteria, she received the diagnosis of DCD. At that time, head circumference was not measured. She was also evaluated by the ophthalmologist because of pseudostrabismus of her left eye, but no other visual impairments or papilledema were observed. Sometimes, she complained of having headaches, especially after a busy social day, but she did not show other signs of elevated intracranial pressure. At the age of 7 years, she experienced generalized status epilepticus. A brain computed tomography (CT) was then performed, which showed hydrocephalus without evident signs of parenchymal damage. After remission, she was referred to the outpatient clinic of pediatric neurology at the UMCG for further diagnostic investigations. The parents reported progressive cognitive problems after the status epilepticus, mainly affecting memory and automaticity. Neurological examination of the cranial nerves showed mild overshoot saccades and mild esotropia of the left eye, but no papilledema. A stiff walking pattern was observed, which increased when walking on toes and heels, along with impaired tandem gait, mild hypertonia in the legs, and somewhat brisk patellar and ankle jerk reflex bilaterally, with nonsustained clonus. There was frontal bossing. Head circumference measured 55.0 cm (+2.25 SD; Figure 1). Because of the increase in head circumference compared to the last measurement at the age of 15 months, there was a suspicion of an acquired cause for the hydrocephalus. Brain magnetic resonance imaging (MRI) confirmed the results of the CT, showing hydrocephalus involving the lateral, third, and fourth ventricles, as a result of an arachnoid cyst in the interpeduncular cistern. There was a pressure effect on the pituitary stalk, mammillary bodies, optic tract, and optic chiasm (Figure 2). Considering these findings, the diagnosis of DCD was withdrawn. In consultation with the neurosurgeon and the family, it was decided to perform an endoscopic fenestration of the intraventricular cyst.

## Discussion

The patient reported in this article seemed to initially fulfill the DCD criteria, but her phenotype was eventually explained by acquired hydrocephalus caused by an arachnoid cyst in the interpeduncular cistern. Possibly, the symptoms were stationary at first as a result of an incomplete or intermittent obstruction of the ventricles caused by the arachnoid cyst, followed by acute decompensation that may have triggered the status epilepticus. Nonetheless, the clinical cue in this case was the secondary macrocephaly. This case is exemplary, as it is the first in literature reporting an underlying hydrocephalus in a patient previously diagnosed with DCD. Furthermore, in line with previous findings, it shows that in children whose phenotypes seemingly fulfill the criteria for DCD, an underlying etiology could still be found )(unpublished data, M. Garofalo et al., 2024). Because anatomical abnormalities mimicking the DCDr phenotype may present with macrocephaly, including hydrocephalus,^3^ it is recommended to investigate the presence of this clinical feature before attributing the symptoms to the diagnosis DCD. In fact, DCD is generally not associated with macrocephaly.^1–3^ Previously, the combination of DCD and macrocephaly has been reported in a patient with Sotos syndrome,^13^ but the DCD diagnosis is questionable in this case because, by definition, the genetic diagnosis of Sotos syndrome rules out DCD.^1–3^ Because head circumference measurements are noninvasive for the patient and effortless for the clinician, we suggest that these should be implemented in the neurological evaluation of children seemingly fulfilling the DCD. To exclude potential clinical progression, longitudinal head circumference measurements are essential. DCD can be accompanied by minor neurological signs.^14^ However, when head circumference measurements show acute or progressive macrocephaly, and/or in the presence of developmental delay or pronounced neurological signs, such as abnormal muscle tone (including hypertonia), marked hyperreflexia, or other symptoms related to an elevated intracranial pressure, performing neuroimaging is recommended to establish the correct diagnosis.^9^

In conclusion, in children with a phenotype resembling DCD, it is important to rule out an underlying hydrocephalus, as this is a treatable cause for the motor impairments. Longitudinal head circumference measurements, along with neuroimaging on clinical indication, can be helpful to timely identify this type of structural brain abnormality.
